# 89 Bridging Cell Biology and Engineering Sciences: Interdisciplinary Team-based Training in Computational Pathology – ERRATUM

**DOI:** 10.1017/cts.2023.553

**Published:** 2023-06-14

**Authors:** Myles Joshua T. Tan, Akshita Gupta, Jamie L. Fermin, Samuel P. Border, Sanjay Jain, John E. Tomaszewski, Yulia A. Levites Strekalova, Pinaki Sarder

The above abstract [1] published with a duplication of Yulia A. Levites Strekalova’s name in the author list, which appeared as “Yulia A. Levites.” This has been removed.

Additionally, the correct author affiliations were missing. The correct author list and subsequent affiliations should appear as follows:

Myles Joshua T. Tan^1*^, Akshita Gupta^2*^, Jamie L. Fermin^1*^, Samuel P. Border^3^, Sanjay Jain^4^, John E. Tomaszewski^5^, Yulia A. Levites Strekalova^6+^, and Pinaki Sarder^7+^

^1^Department of Electrical & Computer Engineering, University of Florida, ^2^Department of Health Outcomes & Biomedical Informatics, University of Florida, ^3^J. Crayton Pruitt Family Department of Biomedical Engineering, University of Florida, ^4^Division of Nephrology, Washington University School of Medicine in St. Louis, ^5^Department of Pathology and Anatomical Sciences, University at Buffalo, ^6^Department of Health Services Research, Management & Policy, University of Florida, ^7^Division of Nephrology, Hypertension & Renal Transplantation, University of Florida

*Equal contribution, ^+^Co-corresponding

The original abstract has been corrected online to rectify these errors.
